# An Ointment Consisting of the Phage Lysin LysGH15 and Apigenin for Decolonization of Methicillin-Resistant *Staphylococcus aureus* from Skin Wounds

**DOI:** 10.3390/v10050244

**Published:** 2018-05-06

**Authors:** Mengjun Cheng, Lei Zhang, Hao Zhang, Xinwei Li, Yanmei Wang, Feifei Xia, Bin Wang, Ruopeng Cai, Zhimin Guo, Yufeng Zhang, Yalu Ji, Changjiang Sun, Xin Feng, Liancheng Lei, Yongjun Yang, Wenyu Han, Jingmin Gu

**Affiliations:** 1 Key Laboratory of Zoonosis Research, Ministry of Education, College of Veterinary Medicine, Jilin University, Xi’an Road 5333#, Changchun 130062, China; mengjun_c@163.com (M.C.); zhanglei222565@126.com (L.Z.); ZhangHao9914@163.com (H.Z.); vetboss@163.com (X.L.); wym01230307@163.com (Y.W.); xiafei0126@126.com (F.X.); terrence0604@gmail.com (B.W.); saycall850@126.com (R.C.); zhangyf324@163.com (Y.Z.); jiyalu120@163.com (Y.J.); schangjiang@126.com (C.S.); xinple@163.com (X.F.); leiliancheng@163.com (L.L.); youngjune@jlu.edu.cn (Y.Y.); hanwy@jlu.edu.cn (W.H.); 2 Animal Science and Technology College, Jilin Agricultural University, Changchun 130117, China; 3 First Hospital of Jilin University, Jilin University, Changchun 130021, China; amily2222@163.com; 4 Jiangsu Co-Innovation Center for the Prevention and Control of Important Animal Infectious Disease and Zoonose, Yangzhou University, Yangzhou 225009, China

**Keywords:** phage lysin, apigenin, ointment, methicillin-resistant *Staphylococcus aureus*, skin infection

## Abstract

*Staphylococcus aureus* (*S. aureus*) is a common and dangerous pathogen that causes various infectious diseases. Skin damage, such as burn wounds, are at high risk of *Staphylococcus aureus* colonization and infection, which increases morbidity and mortality. The phage lysin LysGH15 exhibits highly efficient lytic activity against methicillin-resistant *S. aureus* (MRSA) and methicillin-susceptible *S. aureus* (MSSA) strains. Apigenin (api) significantly decreases haemolysis of rabbit erythrocytes caused by *S. aureus* and shows anti-inflammatory function. LysGH15 and api were added to Aquaphor to form an LysGH15-api-Aquaphor (LAA) ointment. The LAA ointment simultaneously exhibited bactericidal activity against *S. aureus* and inhibited haemolysis. In an LAA-treated mouse model of an MRSA-infected skin wound, the mean bacterial colony count decreased to approximately 10^2^ CFU/mg at 18 h after treatment (and the bacteria became undetectable at 96 h), whereas the mean count in untreated mice was approximately 10^5^ CFU/mg of tissue. The LAA ointment also reduced the levels of pro-inflammatory cytokines (TNF-α, IL-1β, and IFN-γ) and accelerated wound healing in the mouse model. These data demonstrate the potential efficacy of a combination of LysGH15 and api for use as a topical antimicrobial agent against *S. aureus*.

## 1. Introduction

*Staphylococcus aureus* causes an extensive range of infections, from skin to systemic infections, which cause organ failure and death [1,2,3,4]. When the normal skin barrier is disrupted and cell-mediated immunity is suppressed, *S. aureus* can colonize wounds and cause infection [5,6,7,8]. Damaged skin, such as that which occurs in burn wounds, shows a high risk for *S. aureus* colonization and infection, increasing morbidity and mortality among burn patients [6,9,10,11] because the host defence against bacterial infections is severely impaired after burn injury [12,13]. Antibiotics, such as mupirocin, are the most commonly employed antimicrobial agent for skin burn wounds [14,15,16]. However, the increased incidence of antibiotic-resistant bacterial strains, such as methicillin-resistant *Staphylococcus aureus* (MRSA), threaten the efficacy of antimicrobial therapies [7,14].

Endolysins encoded by bacteriophages are capable of lysing host cells by breaking down the cell wall [17,18,19]. Phage lysins such as MV-L [20] and PRF-119 [21,22] may prove effective as alternative strategies for fighting multi-drug resistant bacterial infections. LysGH15, a lysin derived from the staphylococcal phage GH15, has been shown to possess efficient lytic activity against MRSA and methicillin-susceptible *S. aureus* (MSSA), both in vitro and in vivo [17,19]. LysGH15 contains an N-terminal cysteine, histidine-dependent amidohydrolases/peptidases (CHAP) domain, a central amidase-2 domain, and a C-terminal Src homology 3b (SH3b) domain. The individual structures of the three domains of LysGH15 have been determined and the molecular mechanism of its efficient lytic activity have been elucidated [23]. We also discovered that *S. aureus* was unable to develop resistance against LysGH15 under the conditions tested, and that specific anti-LysGH15 antibodies produced in mice are unable to block the activity of LysGH15 [19]. Apigenin (api) is a naturally occurring plant flavonoid that is abundant in various fruits and vegetables [24]. Api has been shown to possess distinct anti-inflammatory and antioxidant activity in chronic inflammation and skin inflammation [25,26,27,28,29,30]. In addition, it has been reported that api inhibits the transcription of the *hla* and *agrA* genes that encode alpha-hemolysin (Hla), which ultimately reduces the production of alpha-hemolysin in *S. aureus* [31]. In a previous study, we determined that the combination therapy of LysGH15 and api exhibited therapeutic potential for treating pneumonia caused by MRSA [18]. However, the effect of LysGH15 and/or api on skin infections caused by *S. aureus* has not been determined yet. Thus, in this study, api was selected. 

In this study, we analysed the effect of an ointment consisting of a combination of LysGH15 and api on the skin decolonization of MRSA. The ointment could not only remove MRSA from the skin wound but also decrease skin inflammation and accelerate wound healing.

## 2. Materials and Methods

### 2.1. Ethics Statement

Female specific-pathogen-free BALB/c mice that weighed 20–22 g (purchased from the Experimental Animal Centre of Jilin University, Changchun, China) were housed in filter-top cages in an air-conditioned animal facility at the National Experimental Teaching Demonstration Centre of Jilin University (Changchun, China). Water and normal mouse chow were provided *ad libitum*, and the mice were monitored daily. All the animal experimental procedures were performed in strict accordance with the Regulations for the Administration of Affairs Concerning Experimental Animals, which were approved by the State Council of the People’s Republic of China (1.11.1988) and the Animal Welfare and Research Ethics Committee at Jilin University. The permission code was 201701002 and the permission date was 5, Janaury, 2017.

### 2.2. Bacterial Strains

The community-associated MRSA strain USA300-TCH1516 (USA300) (ATCC^®^ BAA-1717™) was obtained from the American Type Culture Collection (ATCC, Manassas, VA, United States) and employed throughout this study. USA300 was routinely grown in brain heart infusion (BHI) broth (Becton, Dickinson and Company, Franklin Lakes, NJ, USA) at 37 °C with shaking at 200 revolutions per minute (rpm). 

### 2.3. LysGH15-Api-Aquaphor Ointment Preparation

An *Escherichia coli* BL21 (DE3) strain that expressed the full-length LysGH15 protein was previously constructed in the laboratory, and LysGH15 was expressed and purified as described in a previous report [23]. Api was purchased from the National Institutes for Food and Drug Control (Beijing, China) [17,18]. LysGH15 and api were incorporated into commercially prepared Aquaphor (Eucerin, Hamburg, Germany), which is an emollient ointment. The active ingredients of Aquaphor include petrolatum, mineral oil, ceresin, lanolin alcohol, panthenol, glycerine, and bisabolol [32]. 

### 2.4. In Vitro Activity of LysGH15-Api-Aquaphor Ointment

We performed a plate lytic assay to determine the efficacy of the LysGH15-api-Aquaphor ointment. The MRSA strain USA300 was grown to the exponential growth phase (with an optical density at 600 nm (OD_600_) of 0.6) in BHI broth at 37 °C, with shaking at 200 rpm. The bacteria were collected and washed three times (5000× *g* for 1 min at 4 °C) with phosphate-buffered saline (PBS) [18,19]. The culture of USA300 (1 × 10^7^ CFU) was spread onto BHI agar. The following ointment formulations were prepared, using the ULTRA-TURRAX disperser (IKA, Staufen, Germany) to mix them. Aquaphor containing LysGH15 (60 μg per 0.1 g ointment) and api (500 μg per 0.1 g ointment), Aquaphor containing LysGH15 (60 μg per 0.1 g ointment), Aquaphor containing api (500 μg per 0.1 g ointment), pure api (100 μg per 100 μL PBS), pure LysGH15 (120 μg per 100 μL PBS), pure Aquaphor (0.1 g), and the commercially available topical ointment mupirocin (2%, 0.05 mg) [32] were used. The antibacterial activity of each formulation against USA300 was detected by dripping each ointment onto BHI plates, followed by incubation at 37 °C. The plates were observed and imaged after 12 h.

The bactericidal activities of different formulations were determined by using time kill assay in vitro. The LAA ointment consisted of lyophilized LysGH15 (50 µg LysGH15 per 0.1 g ointment) and api (500 μg api per 0.1 g ointment) mixed with Aquaphor. The LysGH15-Aquaphor (LA) ointment consisted of LysGH15 (50 µg LysGH15 per 0.1 g ointment) mixed with Aquaphor. The api-Aquaphor (AA) ointment consisted of api (500 μg api per 0.1 g ointment) mixed with Aquaphor. To determine the activity of different formulations in vitro, time kill assays were performed [19]. For the assay, 0.1 g of LAA ointment, LA ointment and AA ointment, pure api (500 μg api per 100 μL PBS), pure LysGH15 (50 µg per 100 μL PBS), pure Aquaphor, and mupirocin (2%, 0.05 mg) were added to USA300 (1 × 10^7^ CFU) cultures and incubated at 37 °C [32]. The CFUs associated with each formulation was counted at 10 min by diluting and plating onto BHI agar at 37 °C. The experiments were repeated thrice.

Scanning electron microscopy (SEM) was performed to assess the activity of the different formulations in vitro*.* USA300 was grown to the exponential growth phase (OD_600_ of 0.6) in BHI broth at 37 °C, with shaking at 200 rpm. The bacteria were collected and washed three times (5000× *g* for 1 min at 4 °C) with PBS. The different formulations were separately added to the USA300 suspensions (1 × 10^7^ CFU). The bacterial lysates were harvested by centrifugation (1100× *g* for 1 min) at 0, 1, or 2 min after treatment. Subsequently, the bacterial lysates were fixed with glutaraldehyde and dehydrated in a graded ethanol series for 20 min. The specimens were dried overnight at room temperature. The dried and frozen samples were prepared for SEM analysis (Hitachi S-3400N, Hitachi High-Technologies Europe GmbH, Krefeld, Germany) [33].

Haemolytic activity was assessed using rabbit erythrocytes, as previously described [34]. The different formulations (LAA, AA, api, Aquaphor, LAA with undiluted anti-LAA serum, and AA with undiluted anti-LAA serum) were added to USA300 cultures (5 × 10^4^ CFU/mL). The turbidity of the bacterial culture solutions were measured at 600 nm at 10 h after culturing (to an OD_600_ of 2.5), and the supernatants were harvested via centrifugation (5500× *g* for 5 min at 4 °C) and then filtered. To achieve a final volume of 1 mL, 100 μL supernatant and a 25 μL red blood cell pellet were added to the PBS. The mixture was incubated for 30 min at 37 °C. Subsequently, unlysed blood cells were removed by centrifugation (5500× *g* for 1 min at 4 °C). After centrifugation, the OD_450_ of the supernatant was measured. The supernatants of the pure USA300 culture served as the 100% haemolysis control [18,31].

### 2.5. Mouse Model of Skin Damage and Topical Treatment

A mouse model of skin damage was established using the female BALB/c mice according to the methods used in a previous study, with some modifications [6]. The mice were anaesthetized by intraperitoneal administration of ketamine (100 mg/kg) and xylazine (10 mg/kg). The hair on an approximately 2-cm^2^ section of the dorsum of each mouse was shaved and cleansed with 70% alcohol, and the epidermis was then stripped using autoclave tape to induce skin damage [32]. The damaged skin was topically colonized with the USA300 cultures, using 10 μL (1 × 10^7^ CFU) suspended in PBS. All the animals were housed individually to prevent post-wounding traumatic damage to the wounds by other mice.

At 24 h after colonization, the mice were randomly separated into six groups: the PBS, Aquaphor, AA, LA, LAA, and mupirocin groups. Each group contained 6 mice. The 0.1 g dose of ointments and commercial mupirocin were applied to the wounds according to the previous study method, with some modification [32]. The mice were observed daily and, then, the wounds were photographed at 1, 3, 5, 7 and 9 days after treatment. The width of each wound and the distance traversed by the epithelium were measured. Subsequently, the percentage of re-epithelialization was calculated [35,36].

After the different formulations were administered and allowed to remain on the USA300-challenged skin for 18 h, 6 mice from each group were randomly selected for euthanasia with an injection of Fatal Plus (sodium pentobarbital) (Sigma, St. Louis, MO, USA) and each wound area was excised and weighed to ensure uniformity among the samples. The skin samples were suspended in filter-sterilized PBS and homogenized with sterile mortars and motor-driven Teflon pestles (Kimble, Chicago, IL, USA) on ice. The bacterial loads in the skin sample homogenates were measured by serial dilution and plating on mannitol salt agar plates (Oxoid, Basingstoke, UK). The concentrations of cytokines (TNF-α, IL-1β, and IFN-γ) in the skin samples were quantified using an enzyme-linked immunosorbent assay (ELISA) (eBioscience, San Diego, CA, USA) according to the manufacturer’s instructions [19].

### 2.6. Histology

At 5 d after injury, one mouse from each of the six groups (PBS, Aquaphor, AA, LA, LAA, and mupirocin groups) was randomly selected, and the wound specimens from each group were harvested and fixed in 4% formaldehyde buffered with PBS. The formalin-fixed tissues were then processed and stained with haematoxylin and eosin (H&E) using a routine staining procedure and subsequently analysed using microscopy [32,37]. 

### 2.7. Quantitative Real-Time Polymerase Chain Reaction (qRT-PCR)

It has been reported that the alternatively activated macrophage (AAM) plays an important role in wound healing. Arginase I and YM1 were identified as the markers of AAM activation [38]. Thus, qRT-PCR was employed to detect the mRNA levels of arginase I and YM1 at 5 d after injury [35]. RNA extraction and reverse transcription was performed with a TaKaRa RT reagent kit using gDNA Eraser (Perfect Real Time, lot no. RR047A, Takara Biomedical Technology (Beijing) Co., Ltd., Beijing, China), according to the manufacturer’s instructions. The resultant cDNAs were amplified using the SYBR Green qPCR kit (Invitrogen, Carlsbad, CA, USA) using the following specific sets of primers: Arginase I (forward 5′-GTC TGG CAG TTG GAA GCA TC-3′, reverse 5′-TGG TTG TCA GGG GAG TGT TG-3′), YM1 (forward 5′-CAT GAG CAA GAC TTG CGT GAC-3′, reverse 5′-GGT CCA AAC TTC CAT CCT CCA-3′), and glyceraldehyde 3-phosphate dehydrogenase (GAPDH; forward 5′-TTC ACC ATG GAG AAG GC-3′, reverse 5′-GGC ATG GAC TGT GGT CAT GA-3′).

### 2.8. Immunological Assays

BALB/c mice were shaved, tape-stripped, and treated daily with LAA or PBS for 9 d. At 10 d, serum samples from the LAA-treated mice were obtained. Each group contained 6 mice. Blood samples were also obtained from the LAA-treated mice for the detection of antibodies using Western blotting. The titre of antibodies against LysGH15 was determined using an ELISA [19,32]. To determine whether the serum samples collected over the 9-d LAA treatment period were able to interfere with the lytic activity and haemolytic inhibition activity of LAA, a neutralization assay was performed [19]. Accordingly, 20 mg of LAA was mixed with 80 μL of undiluted serum collected from the LAA-treated or PBS-treated mice, followed by incubation at 37 °C for 10 min or 1 h. Subsequently, the mixture was added to USA300 (100 μL, 1 × 10^7^ CFU/mL) and further incubated at 37 °C. The CFUs were counted at various time points.

### 2.9. Data Analysis

GraphPad Prism 5 (GraphPad Software, Inc., La Jolla, CA, USA) was utilized to analyse the data from the ELISAs. SPSS version 13.0 (SPSS, Inc., Chicago, IL, USA) was employed for the statistical analysis of other experimental data using a one-way analysis of variance (ANOVA). A *p-*value < 0.05 was considered to be statistically significant. The error bars in the figures represent the standard deviations of the means.

## 3. Results

### 3.1. In Vitro Lytic Activity and Haemolysis Inhibition by LAA

**Figure 1:**
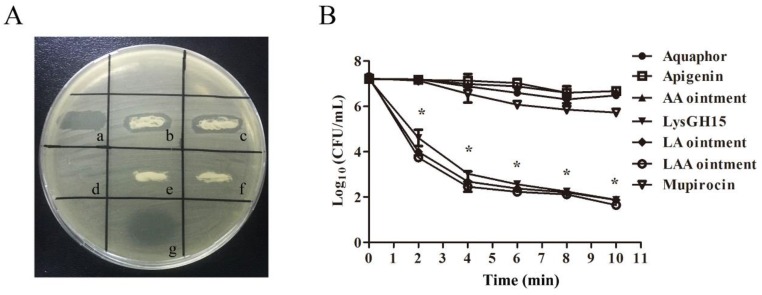
In vitro efficiency of LAA ointment. (**A**) Plate lytic assay. The antibacterial activity of the different ointments against the MRSA strain USA300 was tested using plate assays. The antibacterial rings were measured for the following formulations: the (a) LysGH15 solution (120 μg per 100 μL PBS), (b) Aquaphor containing LysGH15 (600 μg per 1 g ointment), (c) Aquaphor containing both LysGH15 (600 μg per 1 g ointment) and api (5000 μg per 1 g ointment), (d), api solution (100 μg per 100 μL PBS), (e) Aquaphor containing api (5000 μg per 1 g ointment), (f) pure Aquaphor (0.1 g was dripped on BHI plates), and (g) mupirocin (0.05 mg) dripped on the plates. (**B**) Time kill assay. The following formulations were added to cultures of the MRSA strain USA300: LAA, LA, AA, api, LysGH15, mupirocin, and Aquaphor. CFU numbers were counted at different time points as indicated (*n* = 3). The experimental data were analysed by using a one-way analysis of variance. * p < 0.05 compared with the buffer control. The data are representative of three experiments.

**Figure 2:**
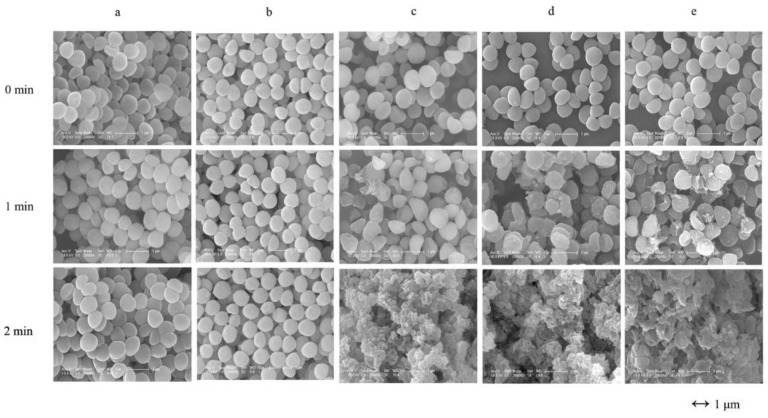
SEM observations of the highly efficient lytic activity of LAA. USA300 was treated with the following formulations: (**a**) Aquaphor, (**b**) api solution, (**c**) LysGH15 solution, (**d**) LA ointment, and (**e**) LAA ointment. The images were obtained by SEM. The bars indicate 1 μm.

To test the bactericidal activity of LAA in vitro, both a plate lytic assay and time kill assay were performed. The plate lytic assay demonstrated that LysGH15-api-Aquaphor and LysGH15-Aquaphor ointment or mupirocin ointment can efficiently inhibit the growth of USA300 and induce distinct halos. AA, Aquaphor, and api exhibited no lytic activity (Figure 1a). When LAA or LA were incubated with USA300, the colony count decreased by 5.2 log units at 10 min after treatment, which was not significantly different compared with the effect of pure LysGH15, as shown in Figure 1b. The AA, Aquaphor, api, or mupirocin groups did not exhibit a decrease in the colony count. It has been reported that mupirocin suppresses growth by interfering with charging of tRNA-Ile (and therefore protein synthesis), whereas LysGH15 lyses cells. The efficient lytic activity of LAA and LA against USA300 was observed by SEM (Figure 2). At 1 min after treatment with LAA, LA, or pure LysGH15, the morphology of the USA300 cells changed, with the cells appearing cracked compared with their normal morphology. Only bacterial debris was observed at 2 min after treatment with LAA and LA, which is similar to the observation for the cells treated with pure LysGH15. The bactericidal activity of LysGH15 was thus not affected by other ingredients in the ointment.

**Figure 3:**
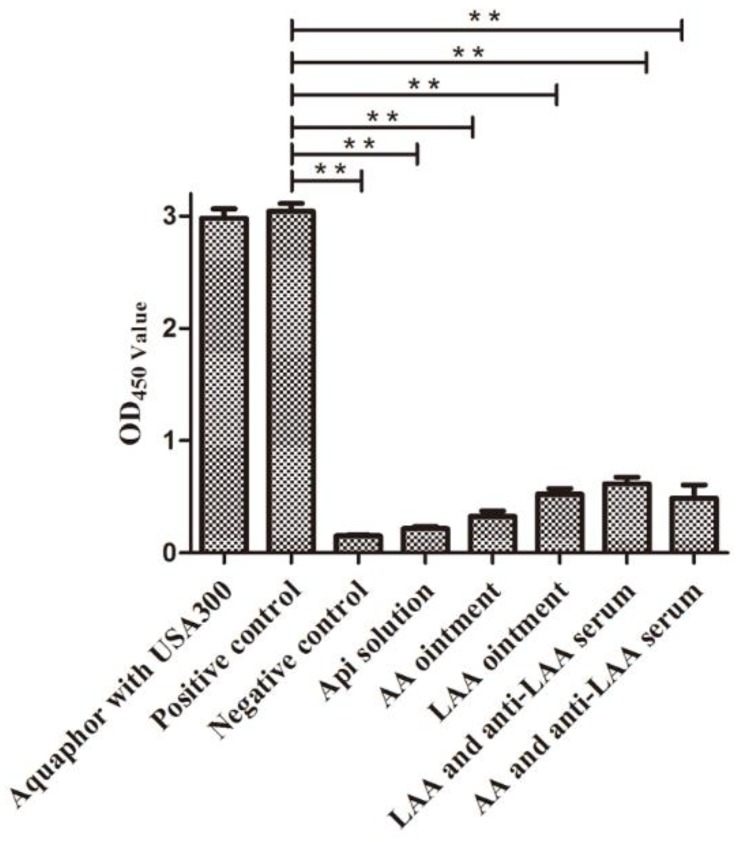
Haemolytic assays. The OD_450_ of the different supernatants was measured. The negative control was PBS-treated erythrocyte suspensions. The experimental data were analysed by using a one-way analysis of variance. ** *p* < 0.01 compared with the positive control. The data are representative of three experiments.

The USA300 culture supernatant, which contains haemolysins, induced haemolysis of rabbit erythrocytes. When Aquaphor was added to the USA300 culture, the haemolytic activity was unaffected. However, when api, AA, LAA, LAA with anti-LAA serum, or AA with anti-LAA serum was added to the rabbit erythrocytes, no haemolysis was detected, as shown in Figure 3. The anti-haemolysis activity of api remained in the ointment.

### 3.2. LAA Accelerates Skin Wound Healing

**Figure 4:**
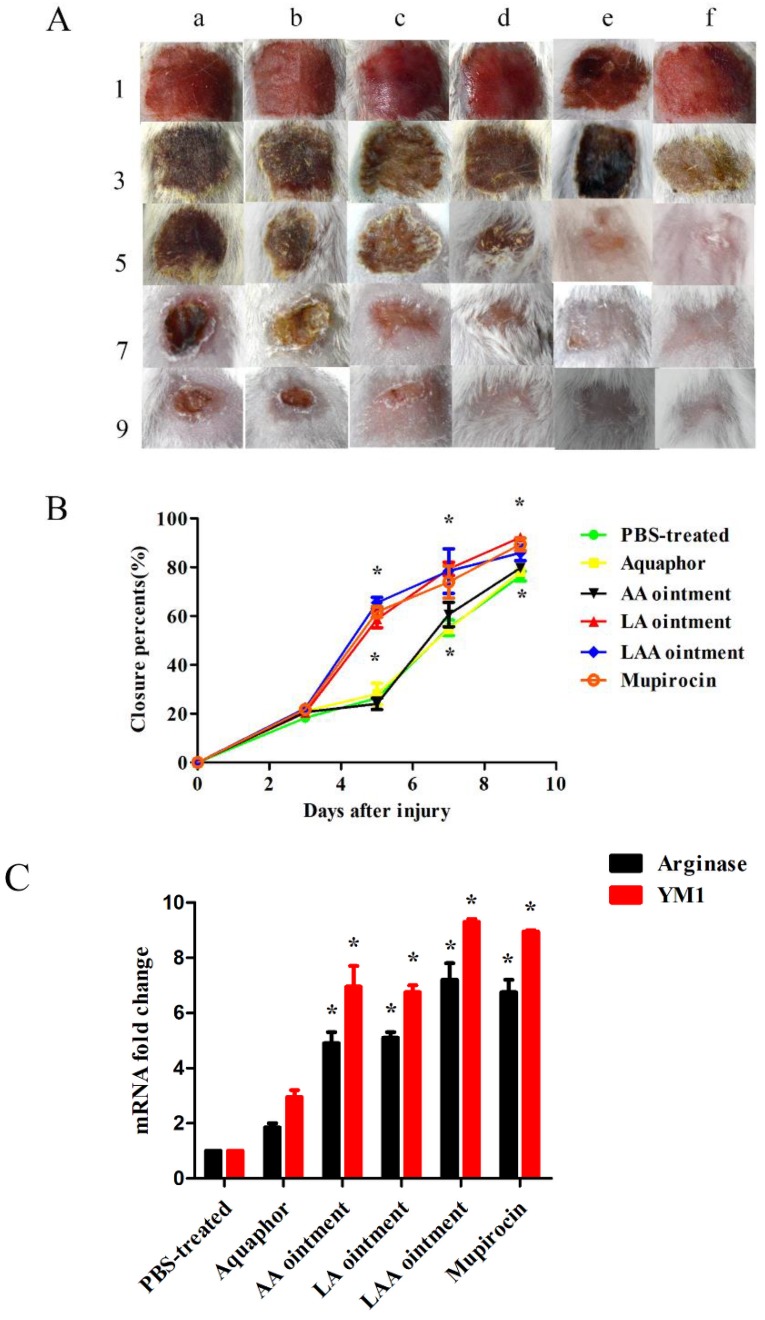
LAA promotes skin wound healing. The shaved and tape-stripped mice were infected with USA300 (1 × 10^7^ CFU/mouse). At 24 h after colonization, the mice were topically treated with different formulations. (**A**) Photographs of wound sites treated with different formulations: the (a) PBS-treated group, (b) Aquaphor-treated group, (c) AA ointment-treated group, (d) LA ointment-treated group, (e) LAA ointment-treated group, and (f) mupirocin-treated group. After treatment, the wound sites were photographed at the 1, 3, 5, 7 and 9 days. (**B**) Wound healing rates. Percentage wound closure in the groups treated with different formulations: PBS-treated, Aquaphor-treated, AA ointment-treated, LA ointment-treated, LAA ointment-treated, and mupirocin-treated. The experimental data were analysed by using a one-way analysis of variance. * p < 0.05 compared with the PBS-treated control. (**C**) Levels of mRNA relative to GAPDH for the AAM-associated genes arginase I and YM1 at 5 d after injury. Alternatively activated macrophages (AAMs) were induced in the skin wounds of LAA-treated mice at 5 d after treatment (*n* = 6 mice per group per experiment). The experimental data was analysed by using a one-way analysis of variance. * p < 0.05 compared with the PBS-treated control.

When the shaved and tape-stripped area of the mice was topically colonized with 1 × 10^7^ CFU USA300, the colony count was 2 × 10^5^ CFU per mg of skin after the challenge, and the skin wound became red and swollen with minimal bleeding and no skin necrosis. Aquaphor, AA, LA, LAA, and mupirocin were used to treat the USA300 infection in the mouse model of skin damage, with PBS being used as the control. As shown in Figure 4a, wound closure was accelerated in the LAA, LA, and mupirocin groups compared with the Aquaphor or AA groups. As shown in Figure 4b, at 5 d, the wound areas shrank by 65.46% in the LAA group, 58.74% in the LA group, and 61.67% in the mupirocin group, compared with 26.34% in the Aquaphor group and 24.05% in the AA group. At 9 d, all skin wounds areas shrank by 95.8% after treatment with LAA, LA, or mupirocin. At 12 d, the mice from the control groups underwent similar wound healing (98%). This observation suggests that LAA and LA accelerated wound closure and subsequent wound healing. LysGH15 plays a key role here but not apigenin. Since AAMs play an important role in wound healing, the transcriptional levels of AAM-associated markers arginase I and YM1 were measured. As shown in Figure 4c, the qRT-PCR results revealed an increase in AAM-associated markers (arginase I and YM1) in the LAA, LA, AA, or mupirocin groups at 5 d after injury compared with the PBS-treated mice. The LAA group showed the highest transcription level of arginase I and YM1 genes compared with the other groups, but the differences with the mupirocin group were not significant.

**Figure 5:**
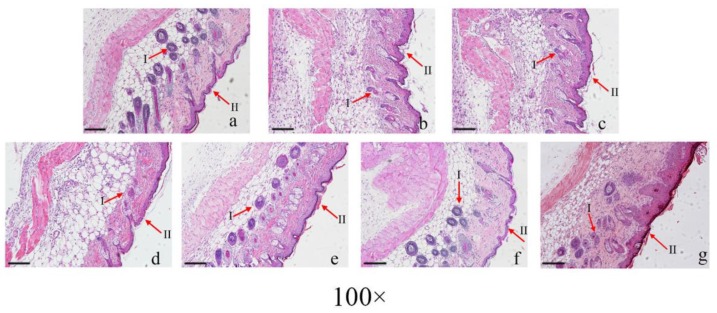
Histological re-epithelialization of skin wounds. The mice were monitored and treated. (**a**) normal mouse skin, (**b**) PBS-treated mouse skin, (**c**) Aquaphor-treated mouse skin, (**d**) AA-treated mouse skin, (**e**) LA-treated mouse skin, (**f**) LAA-treated mouse skin, and (**g**) mupirocin-treated mouse skin. *n* = 6 mice per group per experiment. The skin wounds were stained with haematoxylin and eosin (100×). The arrows indicate the hair follicles (I) and the cortical layer (II) of the skin wounds. Scale bars = 500 μm.

Mouse skin sections stained with hematoxylin and eosin (H&E) were used to evaluate the degree of wound healing. As shown in Figure 5, at 5 d after the USA300 challenge, the skin of the PBS-treated mice exhibited inflammatory cell infiltration, necrosis of the majority of the hair follicles, and shrinkage of the cortical layer, with few new blood capillaries due to the infection. However, mice in the LA, LAA, and mupirocin groups appeared to have fewer inflammatory cells and more new blood capillaries than the PBS-treated mice after one treatment. 

**Figure 6:**
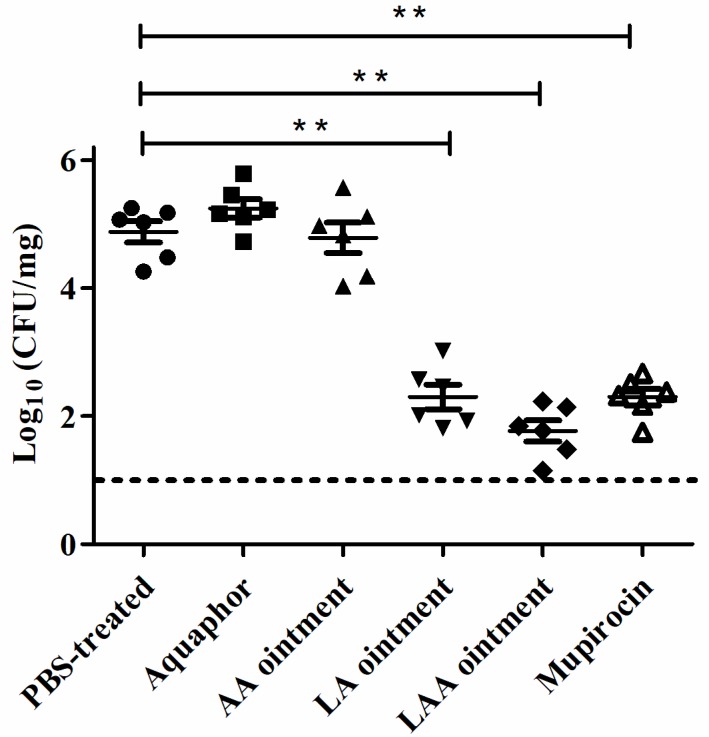
In vivo decolonization activity of LAA in tape-stripped mice infected with MRSA. The tape-stripped mice were infected with MRSA USA300 (1 × 10^7^ CFU/mouse). At 24 h after colonization, the mice were treated with the topical treatments. The bacterial load in the wounded skin at 18 h after treatment was assessed; *n* = 6 mice per group per experiment. The experimental data was analysed by using a one-way analysis of variance. Compared with the PBS-treated group: ** *p* < 0.01. The median value for each group is represented as a horizontal bar, and each sphere represents one mouse. The dotted line indicated the low limit of detection line.

The ability of the different treatments to reduce the bacterial counts in the skin samples was investigated (Figure 6). One dose of LAA resulted in a reduction of 3.3 log units in the number of bacteria (from an initial count of 2 × 10^5^ CFU/mg) at 18 h compared with one dose of AA, PBS, or Aquaphor. The USA300 reduction was lower in the LA group (2.8 log units) than in the LAA group. However, the difference in the efficacy of the dose in the LAA group (3.3 log units reduction) compared with the dose in the mupirocin group (3.1 log units reduction) was not significant. 

**Figure 7:**
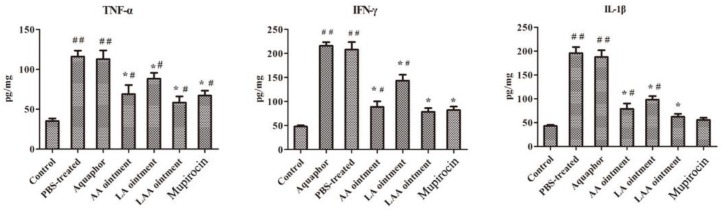
LAA reduced levels of pro-inflammatory cytokines. The levels of TNF-α, IFN-γ, and IL-1β in the excised wound area were determined at 18 h after topical treatment (*n* = 6 mice per group per experiment). The experimental data was analysed by using a one-way analysis of variance. Compared with the control group: ^##^ *p* < 0.01. Compared with the PBS-treated group: * *p* < 0.05. Compared with the control group and PBS-treated group: *^#^ *p* < 0.05.

Since IFN-γ, TNF-α, and IL-1β play a critical role in the pathogenesis of MRSA infections, the levels of these pro-inflammatory cytokines were determined. As shown in Figure 7, the TNF-α, IFN-γ, and IL-1β levels showed similar tendencies. The levels of the three pro-inflammatory cytokines were similar in both the LAA and mupirocin groups and were lower than that of any other treatment group. AA treatment also effectively reduces pro-inflammatory cytokines and was superior to the LA treatment. Thus, the key ingredient here is apigenin but not LysGH15. 

### 3.3. Anti-LAA Serum Did not Affect the Activity of LAA

**Figure 8:**
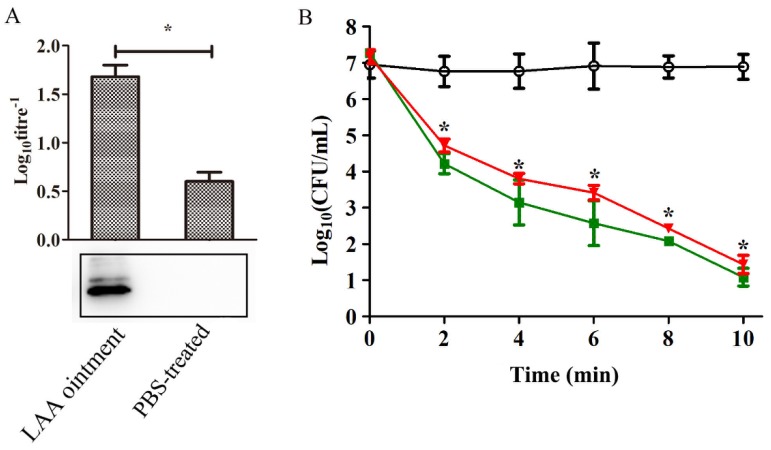
Anti-LAA serum did not neutralize the lytic activity of LAA in vitro. Serum samples from the mice that were exposed to one dose per day of topical LAA for 9 d and the control mice were collected at 10 d after treatment. (**A**) The titres were measured using ELISA. Serum from LAA-treated mice was employed as the primary antibody, and HRP-labelled anti-mouse IgG antibody was employed as the secondary antibody. Western blotting assays of the LysGH15 antibody were carried out (*n* = 6 mice per group per experiment). (**B**) The influence of anti-LAA serum on the lytic activity of LAA. CFUs were counted at different time points, as indicated. Circle: Aquaphor. Box: LAA. Inverted triangle: anti-LAA serum with LAA. *n* = 3 per group per experiment. The experimental data was analysed by using a one-way analysis of variance. * p < 0.05 compared with PBS-treated mice. The data are representative of three experiments.

ELISA and Western blotting assays of the serum samples from the LAA-treated mice revealed the presence of anti-LAA antibodies, with a log10 titre^−1^ of 1.8 (1:64) (Figure 8a). The effect of anti-LAA serum on the bactericidal activity of LAA was tested. As shown in Figure 8b, the bactericidal activity of LAA combined with the anti-LAA serum showed no significant difference compared with that of pure LAA. 

## 4. Discussion

Our study provides the first description of the efficacy of LysGH15-api-Aquaphor (LAA) ointment as a topical treatment in vitro and in vivo. Aquaphor was employed as the ointment base. Aquaphor did not affect the lytic activity of LysGH15 or the haemolysis inhibition of api, and it served as a hydrating agent for the LAA ointment [32]. Thus, this base may enable LysGH15 and api to effectively penetrate into the skin wounds and eradicate the MRSA. 

In vivo, skin wounds were created in mice and subsequently treated with topical applications of different ointments. Both LAA and LA ointments promoted fast re-epithelialization during the nine-day study period, similar to the effect of mupirocin. However, the AA ointment was no better than Aquaphor or PBS. The aetiology of wound healing is complex. According to our results, an important factor in accelerating wound closure and wound healing was the decolonization of the MRSA strain USA300, which was attributed to the lytic activity of LysGH15. Mupirocin is the standard agent for MSSA and MRSA nasal and skin decolonization [39,40]. Mupirocin ointment has also been shown to reduce the risk of catheter-related bacteraemia (relative risk, 0.17; 95% confidence interval, 0.07 to 0.43) [41]. However, it has been reported that the development of mupirocin-resistant *S. aureus* strains was observed after repeated in vitro exposure to the agent after only one week [32]. Mupirocin inhibits the protein synthesis of bacteria by interfering with charging of tRNA-Ile. However, there are many alternative metabolic pathways in bacteria. Thus, it is not unexpected that mupirocin-resistant strains develop very easily. Fortunately, this situation does not apply to lysins. The bactericidal activity of lysins is independent of active host metabolism. In our previous study, no resistant *S. aureus* cells have been recovered after repeated exposure to LysGH15 under the conditions tested [19]. Additionally, as far as we know, resistance has not been identified for any reported lysin [20,32].

The healing process for injured tissues generally involves multiple cytokines that are primarily secreted by immune cells and that are important mediators of host defence, post-injury repair, cell growth, and maturation [35,38,42]. TNF-α, IL-1β, and IFN-γ are multifunctional cytokines that are involved in cell proliferation, inflammation, and immunity at both the local and systemic level. Several studies had shown that these pro-inflammatory cytokines play a critical role in the pathogenesis of MRSA infections and their levels were increased after *S. aureus* infection [38,42,43]. Thus, the levels of TNF-α, IL-1β, and IFN-γ were analysed in this study.

Api is a naturally occurring flavonoid that is considered to be nontoxic and non-mutagenic [44,45]. Api is known to directly suppress Src (a non-receptor tyrosine kinase) activity and subsequently attenuate downstream signalling pathways that are critical for skin inflammation and carcinogenesis [29,44,46]. These findings indicate that api is a potential drug candidate for the treatment of inflammation [24]. Our previous study revealed that combination therapy involving LysGH15 and api exhibits therapeutic potential for treating pneumonia caused by MRSA and reduces the release of cytokines [18]. In the present study, we discovered that the levels of pro-inflammatory cytokines (TNF-α, IL-1β, and IFN-γ) in the skin decreased in infected mice after treatment with LAA or AA. Thus, this effect is attributed to the anti-inflammatory activity of api. 

As an exogenous protein, LysGH15 has the potential to be immunogenic, stimulating the production of antibodies. Our previous study revealed that LysGH15 triggered the generation of LysGH15-specific antibodies in mice when it was subcutaneously administered [19]. However, the antisera did not block the lytic activity nor the binding capacity of LysGH15 against *S. aureus* in vitro or in vivo. We also discovered that LAA can trigger LysGH15-specific antibody production when used to treat wounded skin. However, the titres of antibodies were very low even when LAA was repeatedly administered, as noted in previous research [19,32]

Moreover, the anti-serum did not affect the lytic activity of LAA ointment. In addition, the anti-serum did not affect the haemolysis inhibition exhibited by LAA. No specific antibody against api was detected in this study. Considering the small size of api (similar to conventional antibiotics), this finding is reasonable. 

It has been proved that the N-terminal CHAP domain and C-terminal SH3b domain are the active regions of LysGH15, and their grooves involving the key residues play important roles in lysing and binding *S. aureus* [17]. Anti-LysGH15 antibody produced by the mice during treatment could not block the grooves of the CHAP and SH3b domains. Thus, the antibodies were the binding antibody rather than the neutralizing antibody. In addition, LysGH15 shows higher affinity for the cell wall than for the anti-LysGH15 antibody [19]. 

Our results provide information on a topical antimicrobial agent that works against staphylococci. Specifically, this study provides evidence that the topical application of LysGH15 and api has significant potential as an alternative strategy for MRSA infections.

## Abbreviations

*S. aureus*	*Staphylococcus aureus*
MRSA	methicillin-resistant *Staphylococcus aureus*
MSSA	methicillin-susceptible *Staphylococcus aureus*
CHAP	cysteine, histidine-dependent amidohydrolases/peptidases
SH3b	Src homology 3b
Api	Apigenin
ATCC	American Type Culture Collection
BHI	brain heart infusion
PBS	phosphate-buffered saline
LAA	LysGH15-api-Aquaphor
LA	LysGH15-Aquaphor
AA	Api-Aquaphor
SEM	Scanning electron microscopy
AAM	alternatively activated macrophages
ELISA	enzyme-linked immunosorbent assay
H&E	hematoxylin and eosin
